# A phytosterol-enriched saw palmetto supercritical CO_2_ extract ameliorates testosterone-induced benign prostatic hyperplasia by regulating the inflammatory and apoptotic proteins in a rat model

**DOI:** 10.1186/s12906-019-2697-z

**Published:** 2019-10-17

**Authors:** Heggar V. Sudeep, Karempudi Venkatakrishna, Ballal Amrutharaj, Anitha, Kodimule Shyamprasad

**Affiliations:** R&D Center for Excellence, Vidya Herbs Pvt. Ltd, Jigani Industrial Area, Anekal Taluk, #14A, KIADB, Jigani I phase, Bangalore, Karnataka 560 105 India

**Keywords:** Prostate enlargement, Inflammation, Cell death, Herbal medicine

## Abstract

**Background:**

Benign prostatic hyperplasia (BPH) is a pathological condition affecting older men. BPH complications often lead to deterioration in the quality of life. *Serenoa repens* (Saw Palmetto) is used for treating lower urinary tract infections in traditional medicine.

**Methods:**

This study was performed to compare the efficacy of β-sitosterol enriched saw palmetto oil (VISPO) and conventional saw palmetto oil (SPO) extracted using supercritical fluid extraction, in alleviating the BPH complications using testosterone-induced BPH model rats. The animals received testosterone (5 mg/kg *s.c.*) with or without SPO and VISPO (200 and 400 mg/kg b.w.) or Finasteride (1 mg/kg b.w.) *p.o.* for 28 days. At the end of the experiment, overnight fasted animals were euthanized, blood samples collected for serum analysis of testosterone. Prostate tissue histomorphology was examined by hematoxylin and eosin (H&E) staining. Western blot analysis was performed using prostate tissue homogenates.

**Results:**

VISPO exhibited superior efficacy compared to SPO as evident from the significant decrease in prostate weight to body weight ratio, serum testosterone level and increase in growth inhibition of prostate tissue compared to BPH group (*p* < 0.001). Histological examination of prostate tissue samples showed that VISPO treatment was comparatively better than SPO in improving the hyperplastic patterns. Further, VISPO significantly regulated the expression of inflammatory and apoptotic marker proteins in BPH rats.

**Conclusion:**

Our data provide experimental evidence that β-sitosterol enriched saw palmetto oil could be higher efficacious in treating the BPH complications compared to the conventional saw palmetto oil preparations.

## Background

Benign prostatic hyperplasia (BPH) is an age-related benign proliferative disease affecting males [1]. BPH is characterised by benign enlargement of prostate deteriorating quality of life in older men [2, 3]. BPH complications have been evident in 20% of the male population in their 50s and the prevalence increased to > 35% in men aging 70 and above [4]. BPH is characterized by the increased proliferation of stromal and glandular epithelial cells resulting in the enlargement of prostate size and weight [5]. Androgens play a key role in the progression of BPH. Conversion of circulating testosterone to dihydrotestosterone (DHT) by 5α-reductase in the prostate mediates BPH via regulation of androgen receptor transcription [6].

Experimental data from previous studies have demonstrated a relationship between inflammation of the prostate and BPH development [7, 8]. Inflammatory infiltrates were detected in the prostate tissue indicating chronic inflammation could be a cause of BPH [9]. Earlier reports also suggest that there occurs an upregulation of anti-apoptotic proteins correlated with the expression of Cox-2 causing a reduction in prostate tissue cell death [10]. Elevated levels of inhibitors of apoptosis proteins (IAPs) have been shown in BPH and prostate cancer [11]. Hence stimulation of apoptosis could be a promising strategy in the treatment of BPH [12].

Current treatments for BPH are based on strategies such as inhibition of 5α-reductase, attenuation of gonadotropin-releasing hormone, and blocking of α-adrenoreceptors. Inhibitors of 5α-reductase suppress the conversion of testosterone into a more potent metabolite, 5α-dihydrotestosterone (DHT) [13]. Alpha blockers and 5α-reductase inhibitors improve the BPH symptom and maximal urine flow rate. However, these are associated with side effects such as erectile dysfunction, decreased libido, and reduced semen count [14, 15]. Herbal medicine for the treatment of BPH and associated complications has drawn attention in the recent past, due to the minimal side effects [16, 17].

*Serenoa repens* commonly known as American dwarf Saw palmetto plant contains bioactive compounds such as phytosterols, fatty acids and their ethyl esters derived from dried fruits [18]. Herbal preparations from Saw palmetto have been used for the treatment of BPH. Saw palmetto products had been evaluated clinically against BPH [19]. The pharmacological effects of saw palmetto extract have been previously studied in experimental animal models of BPH [20]. Oki et al. have reported the effect of saw palmetto extract on urodynamic symptoms and micturition reflux in a rat model [21]. In another study saw palmetto whole berry and extract were demonstrated to have efficacy in influencing BPH by reducing the androgen-induced prostate enlargement [22]. Recently, Pais et al. reported the 5-α reductase inhibitory potential of saw palmetto supercritical CO_2_ extract using a cell-free in vitro test system [23]. The present study was conducted to evaluate the efficacy of VISPO, a novel supercritical CO_2_ extract of saw palmetto fruits with higher β-sitosterol content (3%). The results were compared with the conventional saw palmetto oil (SPO). We have observed higher efficacy of VISPO, alleviating the BPH complications as evident from the in vivo experiments.

## Methods

### Saw palmetto oil preparations

SPO and VISPO supercritical CO_2_ extracts were procured from the Department of Phytochemistry, R&D Centre for Excellence, Vidya Herbs Pvt. Ltd., Bangalore. The samples were reconstituted in 0.1% carboxymethyl cellulose (CMC) for in vivo experiments.

### Chemicals and reagents

β-sitosterol (95%) (Sigma Aldrich), LCMS grade methanol and acetonitrile from J.T. Baker (Philipsburg, NJ, USA), Testosterone (Himedia), Finasteride (Dr. Reddy’s Laboratory, India), Bradford reagent (Bio-Rad). Antibodies against cyclooxygenase 2 (Cox-2), nuclear factor kappa B-p65 (NF-kB-p65), Akt, pAkt, caspase-9, Bcl-2, Bax, β-actin and HRP-conjugated goat IgG antibody, were purchased from Santa Cruz Biotechnology, Inc. (Dallas, TX, USA).

### Liquid chromatography-mass spectrometry (LCMS) analysis

The SPO and VISPO extracts were analyzed for β-sitosterol content by LCMS/MS using Shimadzu triple quadrupole mass spectrometer with Nexera X2 UPLC System (LCMS-8050). The chromatographic separation was achieved using analytical column: Kinetex C18 (2.1 × 150 mm, Phenomenex), temperature 40 °C, the detection wavelength 205 nm. The isocratic elution was carried out with the mobile phase composition methanol: acetonitrile (40:60), flow rate: 0.5 ml/min, injection volume: 5 μl.

### Animals

Forty-two male Wistar rats (10-week-old) were purchased from Biogen, Bangalore, India (CPCSEA Reg. No.971/bc/06). The animals were housed in accordance with the CPCSEA (Committee for the Purpose of Control and Supervision of Experiments on Animals) guidelines. After a one-week acclimatization period the animals were housed in rooms maintained at 22 ± 2 °C and 30–70% humidity. Water and standard pellet diet were given ad libitum. The animal experiments were conducted after due clearance from the Institutional Animal Ethics Committee (IAEC) of Vidya Herbs (P) Ltd. (VHPL/PCL/IAEC/08/18).

### BPH induction and dosage

The animals were divided into seven groups of six rats each. Group I rats received 0.1% CMC in saline and served as normal control. Group II animals (BPH control) were administered with a daily dose of 5 mg/kg testosterone subcutaneously for 28 days. In groups III-VII, BPH was induced in rats using testosterone 5 mg/kg in olive oil subcutaneously and a simultaneous treatment with different doses of SPO, VISPO (200 and 400 mg/kg b.w.) or Finasteride (1 mg/kg b.w.) *p.o.*, for 28 days, respectively. At the end of the treatment period, blood was collected prior to the necropsy. Animals were sacrificed by an overdose of gaseous anesthesia (isoflurane) in an anesthetic chamber. The vital parameters such as prostate weight, prostate weight to body weight ratio, growth inhibition of prostate, serum testosterone level, expression of inflammatory and apoptotic proteins in prostate tissue were evaluated.

### Prostate weight

Prostate tissues were excised, rinsed and weighed immediately after removal. The PW/BW ratio was calculated using the following equation: PW/BW ratio = (Prostate weight of each animal from the experimental group / Body weight of each animal from the experimental group) × 1000. The percentage of growth inhibition was calculated as follows: 100 – [(treated group – control group) / (BPH group – control group)] × 100.

### Determination of testosterone in serum

Blood samples were collected at the end of the experiment; coagulated at room temperature for 20 min. Serum was separated by centrifuging at 3000 g, 4 °C. Serum samples were analyzed for testosterone levels using commercial ELISA kit (582,701; Cayman Chemical Co., Ann Arbor, MI, USA) following the manufacturer’s protocol.

### Histological examination

The prostate tissue samples were fixed in 4% formalin, dehydrated with a graded alcohol series, embedded in paraffin, and then cut into 4 μm thickness. The sections were stained with hematoxylin and eosin (H&E, Sigma-Aldrich, St. Louis, MO, USA). The images were captured using a microscope (Leica, Germany).

### Western blot analysis

The prostatic tissue from each animal was homogenized in lysis buffer and incubated for 20 min to induce cell lysis. Tissue extracts were centrifuged at 12000 rpm for 20 min and the supernatants were transferred to clean tubes. Aliquots of protein samples (30 μg) were resolved on 8–15% sodium dodecyl sulphate-polyacrylamide gel electrophoresis gels and transferred on to a nitrocellulose membrane. The membranes were incubated for 1 h with blocking solution and subsequently incubated with 1:500 dilution of primary antibodies for NF-kB-p65 (sc-8008), Cox-2 (sc-19,999), Akt (sc-56,878), pAkt (sc-514,032), caspase-9 (sc-73,548), Bcl-2 (sc-7382) and Bax (sc-20,067) overnight at 4 °C. The membranes were washed three times with 0.1% Tween 20 in TBS followed by incubation with Horseradish peroxidase-conjugated goat IgG antibody (1:4000 dilution) for 1 h at room temperature. Detection was performed on ImageQuant™ LAS 500 (GE Healthcare Life Sciences). Densitometry analyses of the results were made using Image J software (version 1.46, National Institutes of Health, Bethesda, Maryland). β-actin was used as a loading control.

### Statistical analysis

The results are expressed as mean ± SEM. Comparison between groups was performed using one-way analysis of variance (ANOVA) followed by Dunnet’s t-test. The data were considered statistically significant at *p* < 0.05. The statistical analyses were performed using the GraphPad Prism software version 5.0 (GraphPad Software, Inc., La Jolla, CA, USA).

## Results

### LCMS analysis of saw palmetto oil preparations

**Fig. 1:**
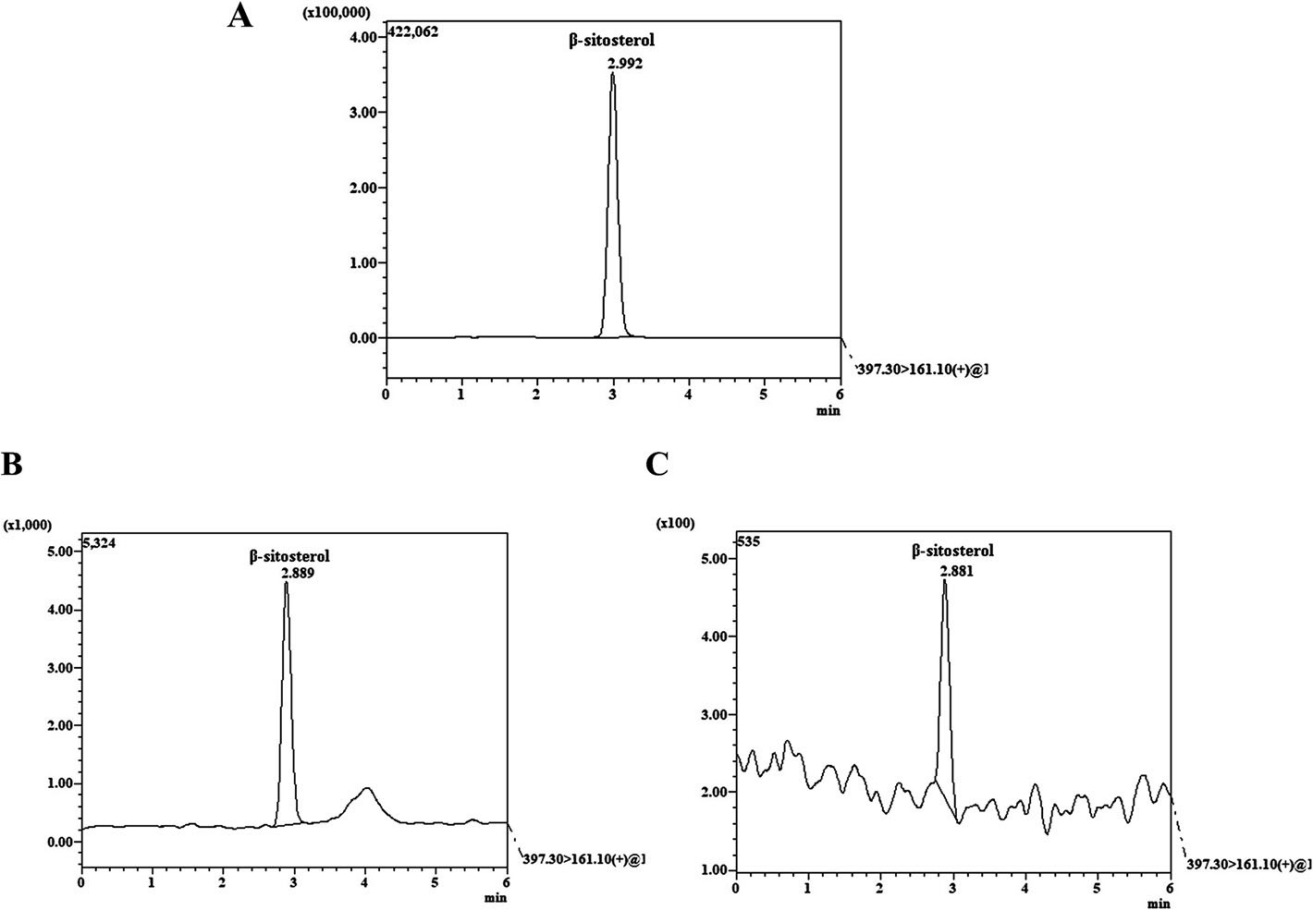
LCMS/MS analysis of saw palmetto extracts. **a** Standard β-sitosterol, **b** VISPO and **c** SPO

The saw palmetto oils were quantified for the β-sitosterol content. LCMS analysis revealed the presence of 0.2 and 3% of β-sitosterol in SPO and VISPO respectively (Fig. 1).

### VISPO prevents hyperplasia in testosterone-induced BPH

**Fig. 2:**
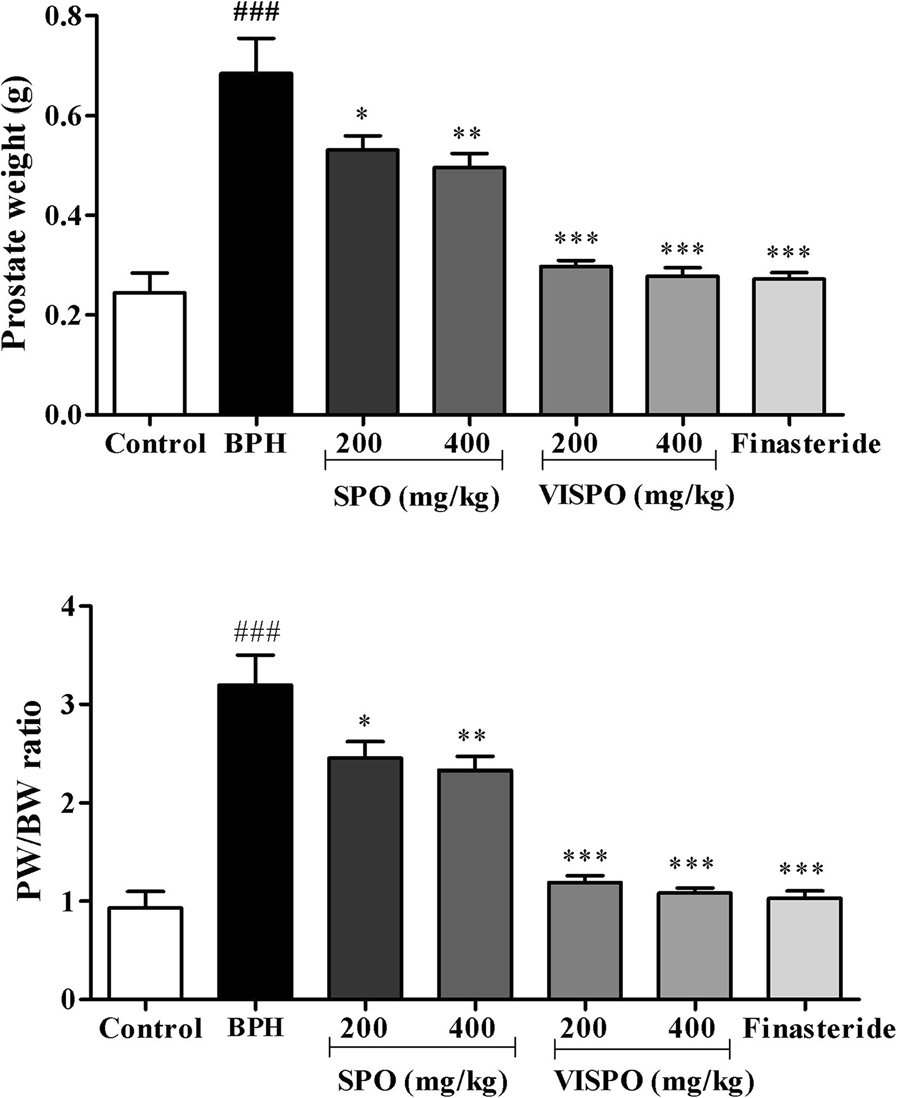
Effect of SPO and VISPO on prostate weight and prostate to body weight (PW/BW) ratio. The data were analyzed by one way ANOVA followed by Dunnet’s t-test. ## *p* < 0.01 vs. control group. * *p* < 0.05, *** *p* < 0.001 vs. the BPH group

The mean prostate weight of animals in the BPH group was significantly increased as compared to the normal rats (*p* < 0.001) indicating that testosterone-induced BPH in rats. The prostate weights were reduced considerably in the Finasteride and VISPO treated groups (*p* < 0.001). There was a dose-dependent decrease in prostate weight in extract treated animals (Fig. 2). The prostate weight in BPH group was 2.83-fold higher than control rats (Table 1). The treated groups exhibited an appreciable reduction in the prostate weight. There was a reduction of 1.28 and 1.38-fold in prostate weight of 200 and 400 mg/kg SPO treated rats respectively. Interestingly, VISPO treated groups markedly decreased the prostate weight compared to BPH group (*p* < 0.001). 400 mg/kg treatment of VISPO reduced the prostate weight by 2.43-fold which was comparable to Finasteride treatment (2.51 fold). The percentage growth inhibition of prostate was 90.9% at 400 mg/kg VISPO treated group. Further, PW/BW ratio in BPH group was significantly increased as compared to control group (*p* < 0.001). Administration of VISPO showed superior efficacy in reducing the PW/BW ratio, as compared to SPO treatment.

**Table 1:** Prostate growth in BPH model rats

Group	Prostate weight (g)	Inhibition of growth (%)
Control	0.24 ± 0.04	–
BPH	0.68 ± 0.07^###^	–
SPO (200 mg/kg)	0.53 ± 0.03^*^	34.09
SPO (400 mg/kg)	0.49 ± 0.03^**^	43.18
VISPO (200 mg/kg)	0.29 ± 0.01^***^	88.63
VISPO (400 mg/kg)	0.28 ± 0.02^***^	90.9
Finasteride (1 mg/kg)	0.27 ± 0.01^***^	93.18

The data were represented as mean ± SEM (*n* = 6). ^###^*p* < 0.001 vs. control group; *p < 0.05, **p < 0.01 and ***p < 0.001 vs. BPH group. Growth inhibition = 100 – ((treated group-control group) / (BPH group-control group) × 100)

*SPO* Saw palmetto oil, *VISPO* Standardized saw palmetto oil

### VISPO restores the serum testosterone level in BPH rats

**Fig. 3:**
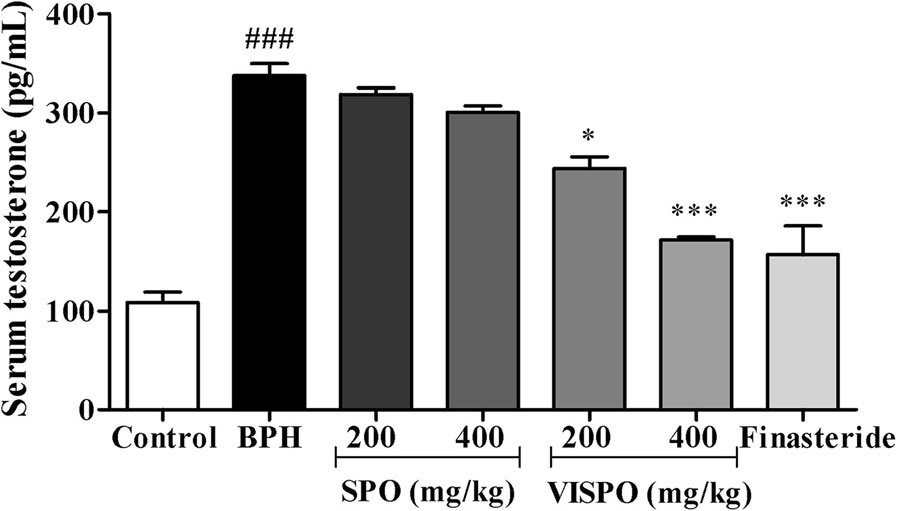
Effect of SPO and VISPO on serum testosterone level. The data were analyzed by one way ANOVA followed by Dunnet’s t-test. ###*p* < 0.001 vs. control group. *p < 0.05, ***p < 0.001 vs. the BPH group

The serum testosterone level was significantly higher in BPH group (337.5 ± 12.15 pg/mL) as compared to control rats (108.6 ± 14.92 pg/mL). As shown in Fig. 3, the level of testosterone was markedly decreased in the treatment groups. The values were highly significant in 400 mg/kg VISPO treated group and comparable to Finasteride (*p* < 0.001).

### VISPO suppressed the hyperplastic patterns in prostate tissue of BPH rats

**Fig. 4:**
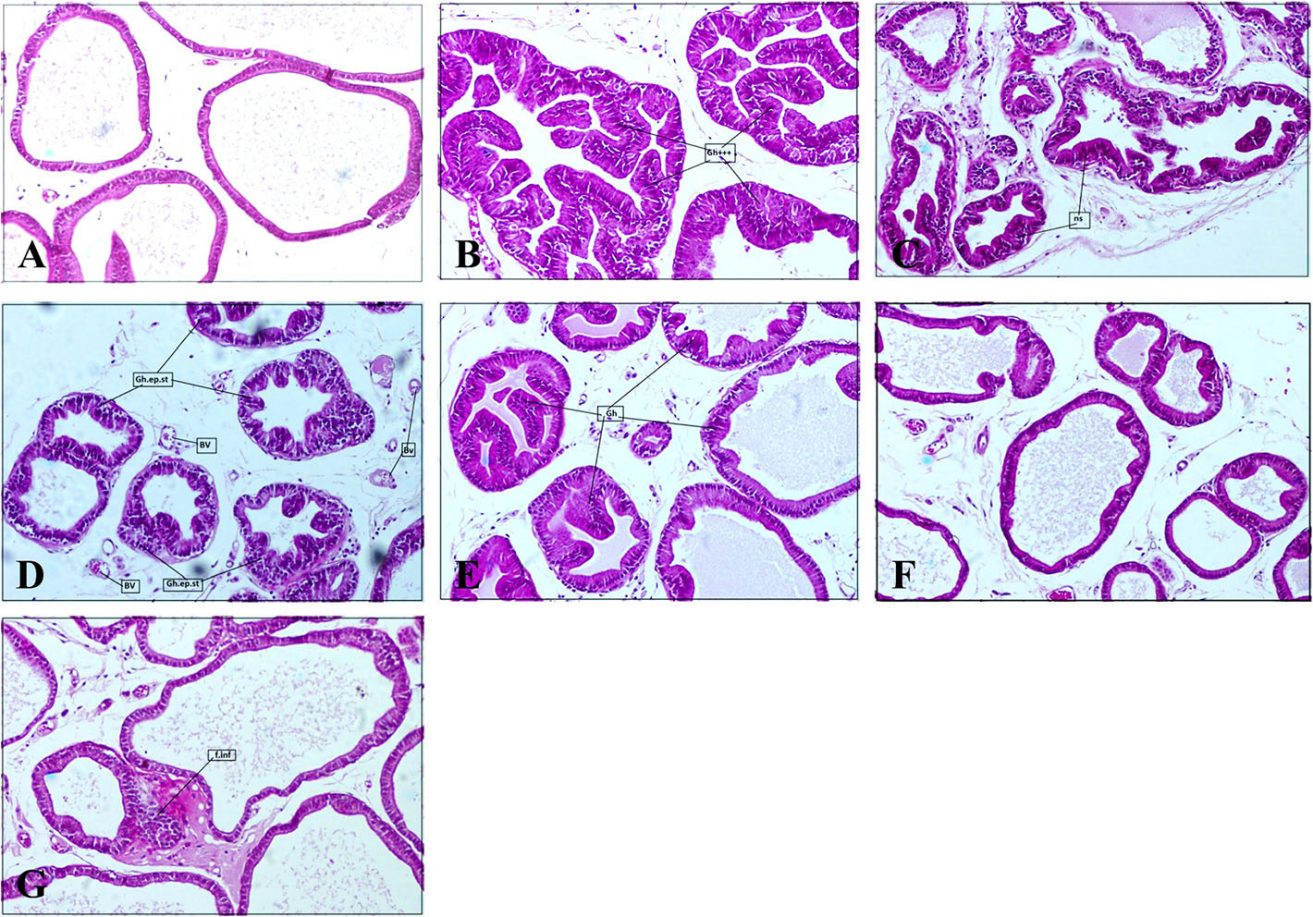
Effects of VISPO administration on prostate hyperplasia. Prostate tissues were fixed, sectioned at 4 μm thickness, stained with hematoxylin and eosin (H&E, magnification, × 100). **a** Vehicle control, **b** BPH/vehicle, **c** BPH/SPO 200 mg/kg, **d** BPH/SPO 400 mg/kg, **e** BPH/VISPO 200 mg/kg, **f** BPH/VISPO 400 mg/kg and **g** BPH/Finasteride 1 mg/kg

Histological examination of prostate tissue revealed that the testosterone treatment induced glandular hyperplasia with decreased glandular luminal area compared to the control animals (Fig. 4b). SPO (200 and 400 mg/kg) and 200 mg/kg VISPO treatment groups exhibited moderate suppression of prostate hyperplasia patterns (Fig. 4c-e). 400 mg/kg VISPO treatment markedly restored the cellular architecture in BPH rats (Fig. 4f). The epithelial cell thickness was reduced, and the luminal area increased considerably. The finasteride group also showed near normal prostatic glands with mild focal inflammation (Fig. 4g).

### VISPO regulated the expression of inflammatory and apoptotic markers in BPH rats

**Fig. 5:**
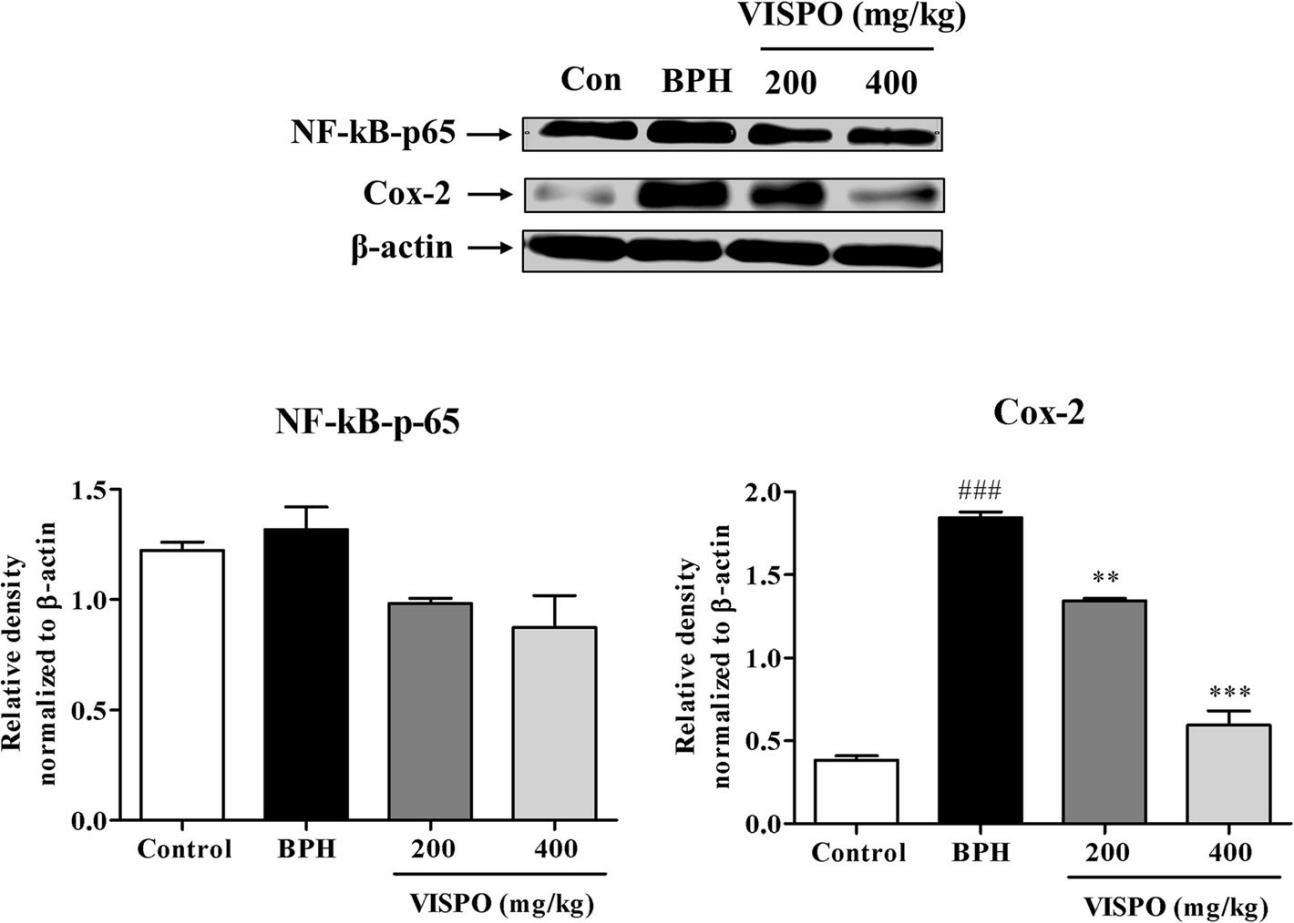
Effect of VISPO administration on the expression of NF-kB and Cox-2 in prostate tissue of BPH model rats. The protein expression was determined by western blotting using specific antibodies. β-actin was used as loading control. Densitometric analysis was performed using Image J software. The data presented as mean ± SEM (###*p* < 0.001 vs. Control group; **p < 0.01 and ***p < 0.001 vs. BPH group)

Testosterone treatment markedly increased the expression of NF-kB (*p* < 0.05) and Cox-2 (*p* < 0.001) in prostate tissue of BPH rats as compared to control group. However, treatment with VISPO significantly down-regulated the expression of these proteins in a dose dependent manner (Fig. 5).

**Fig. 6:**
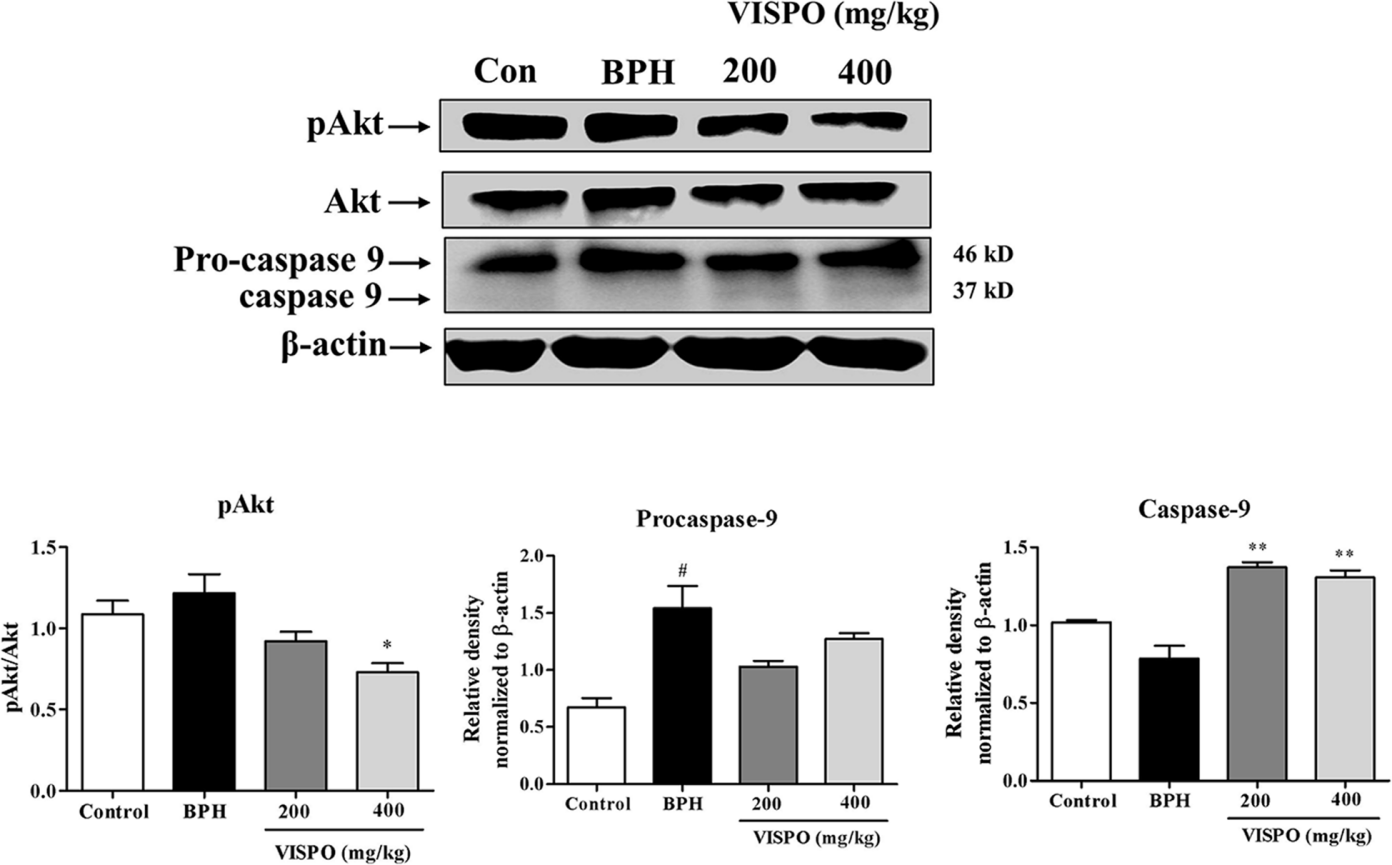
Effect of VISPO administration on the expression of pAkt and caspase-9 in prostate tissue of BPH model rats. The protein expression was determined by western blotting using specific antibodies. β-actin was used as loading control. Densitometric analysis was performed using ImageJ software. The data presented as mean ± SEM (#*p* < 0.05 and ###*p* < 0.001 vs. control group; **p < 0.01 and ***p < 0.001 vs. BPH group)

**Fig. 7:**
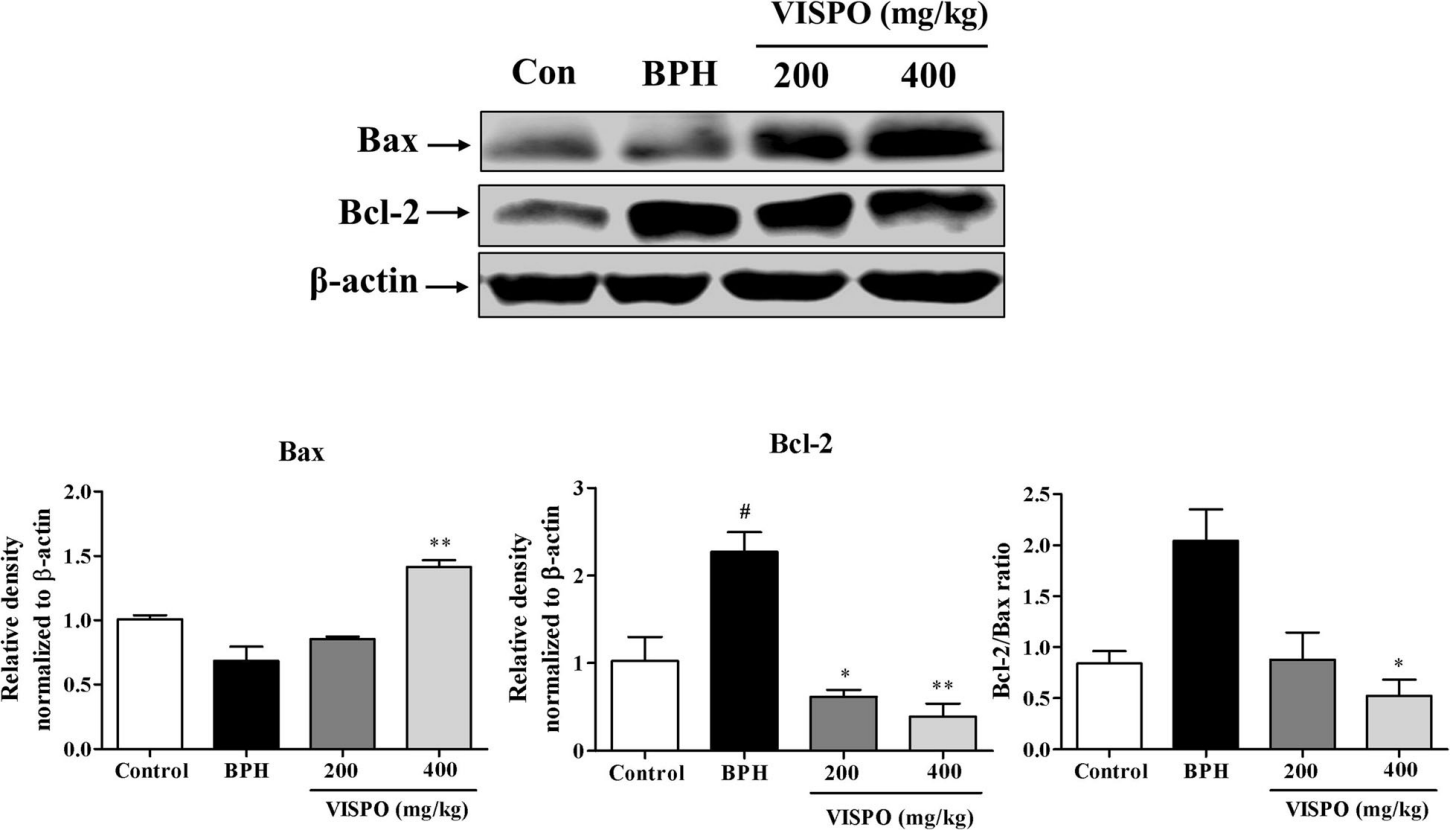
Effect of VISPO administration on the expression of Bcl-2 family in prostate tissue of BPH model rats. The protein expression was determined by western blotting using specific antibodies. β-actin was used as loading control. Densitometric analysis was performed using ImageJ software. The data presented as mean ± SEM (#*p* < 0.05 vs. control group; *p < 0.05 and **p < 0.01 vs. BPH group)

In the prostate tissue of BPH rats there was an obvious increase in the expression of pAkt. Also, VISPO treatment effectively downregulated the phosphorylation of Akt at Ser-473. There was a considerable decrease in the procaspase-9 level in the prostate tissue of BPH rats after treatment with respective doses of VISPO (Fig. 6). Further, VISPO treated groups showed a significant decrease in the antiapoptotic protein Bcl-2, but increased expression of proapoptotic Bax as compared to BPH group (*p* < 0.01) (Fig. 7).

## Discussion

BPH is a common prostate disease affecting older men. It is a major cause of lower urinary tract symptoms deteriorating the quality of life [24]. The BPH pathogenesis has been associated with factors such as genetic predisposition, hormonal imbalance, imbalance in cell proliferation and death and inflammation [25]. The current therapy for BPH involves administration of 5-α reductase inhibitors, phosphodiesterase-5 inhibitors, and laser therapy [13, 26]. Nevertheless, these treatments improve the quality of life some of the patients may feel worse due to side effects of the treatment. For example, Finasteride is a 5-α reductase inhibitor used for treating BPH, effectively alleviating the complication [27]. However, Finasteride is also associated with undesirable side effects such as erectile dysfunction, increased risk of impotency, and ejaculation disorder. This has led to the search for alternative therapies with fewer side effects for the treatment of BPH [28]. A large body of evidence suggest the promising role of phytotherapy for the management of BPH [29].

*S. repens* (Saw palmetto), an evergreen shrub is used long since for the treatment of BPH. Medicinal preparations from saw palmetto, particularly the ripe berries are used to alleviate the symptoms of BPH [30]. It has also been reported that saw palmetto extracts are used to treat prostate cancer in men as alternative medicine [31]. In the present study we have investigated the efficacy of a standardized Saw palmetto oil prepared from supercritical fluid extraction, with higher β-sitosterol content (VISPO) in BPH model rats. The results were compared with conventional saw palmetto oil (SPO).

BPH is primarily characterized by the enlargement of the prostate [32]. In the present study, the testosterone-induced BPH group demonstrated increased prostate weight and PW/BW ratio compared with the control group. A 21 day-treatment of BPH model rats with SPO, VISPO and Finasteride significantly inhibited this increase in prostate weight and PW/BW ratio. It was interesting to observe that VISPO exhibited superior inhibition of prostate weight (*p* < 0.001) compared to SPO. VISPO showed 48% increase in the growth inhibition percentage (90.9%) in comparison to SPO treated group (43.18%) at 400 mg/kg dose. Prostate cell growth in BPH is influenced by the circulating testosterone that acts locally via the production of necessary growth factors [33]. There was a significant decrease in the serum testosterone levels following VISPO administration in BPH rats, comparable to Finasteride treated group. These preliminary results prompted us to further address the biomechanism of VISPO mediated alleviation of BPH.

Experimental data from previous studies suggest that a strong correlation exist between inflammation and BPH. The pro-inflammatory cytokines-induced expression of inflammatory mediators such as Cox-2 and iNOS contribute to prostate enlargement [34]. In agreement with this, we have observed that the Cox-2 levels were increased in the BPH group. However, VISPO treatment dose-dependently decreased the protein level. Further, treatment with 400 mg/kg VISPO significantly downregulated the Cox-2 expression compared to BPH group (*p* < 0.001). It is well known that NF-kB stimulates the expression of proteins contributing to the pathogenesis of inflammation. In fact, activation of NF-kB is a hallmark of inflammation [35]. In the present study, expression of NF-kB in the prostate tissue of BPH rats was considerably increased as compared to control animals. VISPO treatment suppressed the NF-kB expression compared with that of animals in the BPH group. These data suggest possible anti-inflammatory effects of VISPO in the treatment of BPH.

**Fig. 8:**
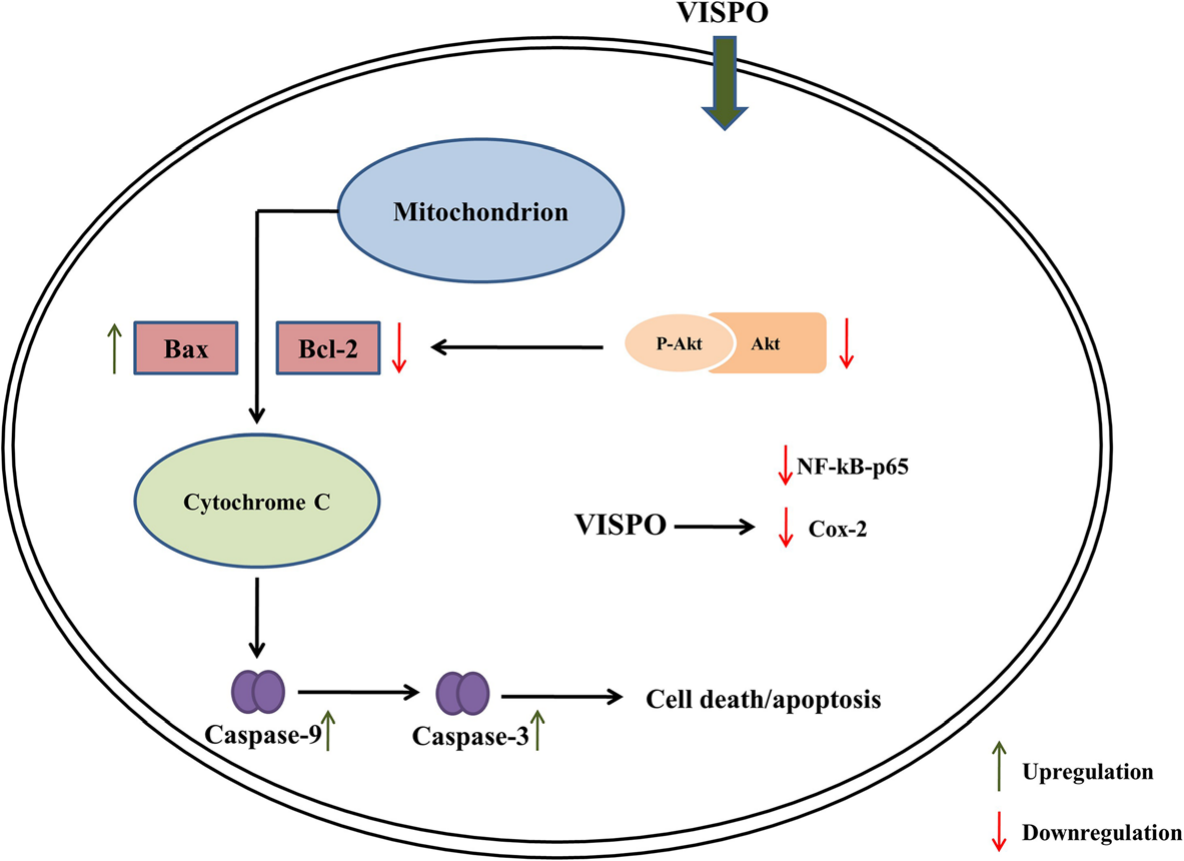
Schematic representation of the proposed mechanism for the efficacy of VISPO in BPH model rats

Activation of Akt has been shown to facilitate cell survival against apoptotic stimuli. Available data from previous literature reveal that activation of Akt up-regulates the expression of anti-apoptotic Bcl-2 through cAMP-response Element-binding protein (CREB) [36]. In agreement to this, we noticed an increase in the expression of pAkt and Bcl-2 in prostate tissue of testosterone-induced BPH rats. However, VISPO treatment induced apoptosis by significantly downregulating these proteins. The pro-apoptotic protein Bax was upregulated by VISPO. There was an appreciable decrease in the ratio of Bcl-2 to Bax following treatment with VISPO. The expression of procaspase-9 was markedly down-regulated by the VISPO treatment compared to BPH control rats. These data suggested that VISPO induced apoptosis through the regulation of Bcl-2 family proteins leading to a caspase-dependent pathway (Fig. 8).

The improved efficacy of the present intervention, VISPO in comparison with conventional saw palmetto oil could be attributed to the higher content of β-sitosterol in the extract. Phytosterols have been well-documented for having anti-inflammatory, antioxidant and anticancer effects [37–39]. Previous studies have reported the potential anti-inflammatory activity of β-sitosterol using in vitro and in vivo models [40, 41]. Enriched content of β-sitosterol in VISPO could possibly contribute to the anti-inflammatory effect in BPH model. Also, in the present study it was found that VISPO exerted the proapoptotic actions in prostate tissue of BPH rats. It has been reported earlier that β-sitosterol induces apoptosis in prostate cancer cells by activating the sphingomyelin cycle [42].

The major pathway for testosterone metabolism in BPH is its conversion into more active form, dihydroxytestosterone (DHT) via 5α- reductase [43]. Cabeza et al., reported the effect of β-sitosterol in BPH as a function of 5α- reductase inhibition [44]. Several clinical studies have previously demonstrated the positive effects of phytosterols (β-sitosterol) on the quality of life in patients with BPH [45, 46]. Clinical data suggest that treatment with β-sitosterol significantly increase the urine flow and decrease the post-void residual volume [47]. Results from our study clearly indicate a plausible role of β-sitosterol for the improvement in the efficacy of saw palmetto oil.

## Conclusion

The β-sitosterol enriched saw palmetto oil was found to be more efficacious than the conventional saw palmetto oil, in preventing the pathogenesis of BPH in rats. The present study for the first time demonstrates the enhancing efficacy of saw palmetto oil attributable to its higher β-sitosterol content. Further clinical interventions pertaining to the safety and efficacy will clearly define the therapeutic potential of VISPO in the treatment of BPH.

## Data Availability

The data sets used and/or analyzed during the current study available from the corresponding author on reasonable request.

## Abbreviations

Akt: Protein Kinase B
ANOVA: Analysis of Variance
Bax: Bcl-2-associated X Protein
Bcl-2: B-cell lymphoma 2
BPH: Benign Prostate Hyperplasia
BW: Body weight
CMC: Carboxymethyl Cellulose
Cox-2: Cyclooxygenase-2
CPCSEA: Committee for the Purpose of Control and Supervision of Experiments on Animals
CREB: cAMP-response Element-binding protein
DHT: Dihydroxytestosterone
ELISA: Enzyme-linked Immunosorbent Assay
H&E: Hematoxylin and Eosin
HRP: Horseradish peroxidase
IAEC: Institutional Animal Ethics Committee
IAP: Inhibitors of Apoptosis Proteins
IgG: Immunoglobulin G
IkB: Inhibitor of Kappa B
iNOS: inducible Nitric Oxide Synthase
LCMS: Liquid Chromatography Mass Spectrometry
NF-kB: Nuclear Factor Kappa-light-chain-enhancer of activated B cells
pAkt: Phosphorylated Protein Kinase B
PW: Prostate weight
SPO: Conventional saw palmetto oil
VISPO: β-sitosterol enriched saw palmetto oil
